# Current status of the working environment of brachytherapy in Japan: a nationwide survey-based analysis focusing on radiotherapy technologists and medical physicists

**DOI:** 10.1093/jrr/rrae082

**Published:** 2024-10-24

**Authors:** Toru Kojima, Hiroyuki Okamoto, Masahiko Kurooka, Naoki Tohyama, Ichiro Tsuruoka, Mikio Nemoto, Kohei Shimomura, Atsushi Myojoyama, Hitoshi Ikushima, Tatsuya Ohno, Hiroshi Ohnishi

**Affiliations:** Department of Radiation Oncology, Saitama Prefectural Cancer Center, 780 Komuro, Ina-machi, Saitama 362-0806, Japan; Division of Radiation Safety and Quality Assurance, National Cancer Center Hospital, 5-1-1 Tsukiji, Chuo-ku, Tokyo 104-0045, Japan; Department of Radiation Therapy, Tokyo Medical University Hospital, 6-7-1 Nishishinjuku, Shinjuku-ku, Tokyo 160-0023 Japan; Department of Radiological Sciences, Komazawa University, 1-23-1 Komazawa, Setagaya-ku, Tokyo 154-8525, Japan; Department of Medical Technology, National Institutes for Quantum Science and Technology, QST Hospital, 4-9-1 Anagawa, Inage-ku, Chiba 263-8555, Japan; Department of Radiotherapy, Jichi Medical University Hospital, 3311-1 Yakushiji, Shimotsuke-shi, Tochigi 329-0498, Japan; Department of Radiological Technology, Faculty of Medical Science, Kyoto College of Medical Science, 1-3 Sonobe-cho oyamahigashi-machi, Nantan-shi, Kyoto 622-0041, Japan; Department of Radiological Science, Graduate School of Human Health Sciences, Tokyo Metropolitan University, 7-2-10 Higashiogu, Arakawa-ku, Tokyo 116-8551, Japan; Institute of Biomedical Sciences, Tokushima University Graduate School, 3-18-15 Kuramoto-cho, Tokushima 770-8503, Japan; Department of Radiation Oncology, Gunma University Graduate School of Medicine, 3-39-22 Showa-machi, Maebashi-shi, Gunma 371-8511, Japan; Department of Radiology, University of Yamanashi Faculty of Medicine, 1110 Shimokato, Chuo-shi, Yamanashi 409-3898, Japan

**Keywords:** brachytherapy, medical physicist, radiotherapy technologist, quality control, image-guided brachytherapy

## Abstract

Brachytherapy (BT), especially in high dose rate (HDR), has become increasingly complex owing to the use of image-guided techniques and the introduction of advanced applicators. Consequently, radiotherapy technologists and medical physicists (RTMPs) require substantial training to enhance their knowledge and technical skills in image-guided brachytherapy. However, the current status of the RTMP workload, individual abilities and quality control (QC) of BT units in Japan remains unclear. To address this issue, we conducted a questionnaire survey from June to August 2022 in all 837 radiation treatment facilities in Japan involving RTMPs. This survey focused on gynecological cancers treated with HDR-BT (GY-HDR) and permanent prostate implantation using low-dose-rate BT (PR-LDR). The responses revealed that the average working time in the overall process for HDR varied: 120 min for intracavitary BT and 180 min for intracavitary BT combined with interstitial BT. The QC implementation rate, in accordance with domestic guidelines, was 65% for GY-HDR and 44% for PR-LDR, which was lower than the 69% observed for external beam radiation therapy (EBRT). Additionally, the implementation rate during regular working hours was low. Even among RTMP working in facilities performing BT, the proportion of those able to perform QC for BT units was ~30% for GY-HDR and <20% for PR-LDR, significantly lower than the 80% achieved for EBRT. This study highlights the vulnerabilities of Japan’s BT unit QC implementation structure. Addressing these issues requires appropriate training of the RTMP staff to safely perform BT tasks and improvements in practical education and training systems.

## INTRODUCTION

Brachytherapy (BT) is a beneficial treatment modality that allows localized high-dose delivery to target tumors while minimizing radiation exposure to normal tissues. High-dose-rate (HDR) BT with a remote afterloading system using ^60^C and ^192^Ir sources for cervical cancer (GY-HDR) and low-dose-rate (LDR) permanent ^125^I seed implantation for prostate cancer (PR-LDR) is one of the standard treatments recommended by Japanese guidelines, respectively [1, 2]. In HDR-BT, 3D image-guided brachytherapy (IGBT) employing computed tomography (CT) and magnetic resonance imaging (MRI) is widely used instead of traditional 2D treatment planning, which utilizes radiographs taken in two directions [3–7]. This allows treatment plans to be designed considering both tumor and organs-at-risk doses, thus providing a more effective BT. Additionally, a combination of intracavitary (IC) and interstitial (IS) techniques, called IC and IS (IC/IS) BT, is performed in 15% of patients with gynecological cancer, and its superior dose distribution contributes to its status as a standard treatment [8].

BT is characterized by a single or a small number of fractions, a high dose per fraction and steep dose gradients. Consequently, even minor errors can significantly affect the radiation dose delivered to the patients. Additionally, BT is also time constrained; the entire irradiation process (from patient anesthesia, applicator insertion, treatment planning, to dose delivery) must be completed within a few hours. This time constraint makes thorough checking by multiple personnel difficult and may lead to errors being overlooked. Significant incidents related to BT have been documented even in Japan. For instance, in the context of GY-HDR, an incident occurred where ~30 mm of the anterior region was irradiated in 100 patients over 5 years [9]. Serious incidents have also occurred involving the HDR source remaining in the patient’s body beyond the planned dwell duration, necessitating manual retraction into the HDR unit [10]. An incident occurred in a case of PR-LDR because the calibration date for ^125^I seeds was mistakenly delayed by 1 week [11]. However, only ~30% of the facilities conducted ^125^I seed strength measurements [12]. Considering that BT involves the manipulation of radioactive sources, concerns exist regarding radiation exposure for both medical staff and the general public.

Ensuring the safe and continuous provision of radiation therapy requires a cohesive team of professionals, including radiation oncologists, medical physicists (MPs) and radiotherapy technologists (RTs), to adhere to international safety standards [13]. Within Japanese radiation therapy departments, the distinct roles of RTs and MPs are not always well defined, as shown in the Materials and Methods section. Additionally, since many MPs have RT certification authorized by the government, the term ‘radiotherapy technologists and medical physicists (RTMPs)’ is used as a generic term to encompass both roles. In BT, as in external beam radiation therapy (EBRT), RTMPs play a crucial role and bear significant responsibility in safely and efficiently introducing advanced radiation therapy techniques into clinical practice. The increasing complexity of GY-HDR techniques is expected to increase the workload for RTMPs involved in the quality control (QC) of BT units and related equipment, as well as quality management of treatment techniques. Ensuring safe BT requires RTMPs to possess advanced knowledge and skills encompassing an understanding of treatment protocols, proficiency in handling radioactive sources and effective emergency response capabilities. Consequently, we need to determine an appropriate staffing level based on the quantitative workload of RTMPs and foster an educational environment to achieve these objectives. The International Atomic Energy Agency (IAEA) and the American Society for Radiation Oncology (ASTRO) offer algorithms for calculating the necessary staffing levels [14, 15]. Domestic guidelines have also reported the facility-specific required number of RTMP personnel [16]. However, these numbers do not reflect the actual workload as they were calculated based only on the number of patients undergoing EBRT and the number of treatment devices or personnel required when installing a new BT treatment technique.

Recent technological advancements in radiation therapy have resulted in increased workload complexity, a trend that is expected to persist. Regular quantitative evaluations and updates are essential to establish appropriate staffing levels [17]. Based on a survey conducted alongside this report, Tohyama *et al*. summarized the workload investigation for EBRT [18], while Hayashi *et al*. addressed education systems and prospects [19]. Additionally, Ikushima *et al*. documented the number of BT cases, working conditions of radiation oncologists and nurses, and resident education in Japan [8]. However, these previous studies did not report any investigation of the working environment of RTMPs in BT.

In this study, we conducted a quantitative survey assessing the working environment of RTMPs, the QC status of treatment units specializing in BT and the clinical abilities of individuals. We aimed to clarify the current status and identify issues related to the workload and individual abilities of RTMPs engaged in BT in Japan. Therefore, our objective was to establish a robust system that provides high-quality and safe BT services to patients in Japan.

## MATERIALS AND METHODS

### Survey samples and processes

A questionnaire survey was conducted between June and July 2022. It was divided into two parts: one assessing the workload and QC implementation status at the facilities and the other exploring the ability of individual RTMPs to perform each task. These were conducted in Japanese and simultaneously with EBRT questionnaires for centralized data analysis and respondent convenience. Summarized results other than the BT can be found in previous studies [18, 19]. For the survey on workload and QC status of facilities, the respondents were asked to enter their answers in an Excel file and upload it to a dedicated website using Google Services (Google, CA, USA). We asked respondents to complete their answers on Google Forms to survey the capabilities of individual RTMPs. A questionnaire was administered to all medical facilities providing radiotherapy in Japan. On condition that the results of the survey be published, a total of 837 eligible radiotherapy facilities were asked to respond to the questionnaire via mail or e-mail. The BT questionnaire was limited to GY-HDR for the HDR and PR-LDR for the LDR. This targeted approach aimed to streamline respondent efforts and minimize response variation by concentrating on cases with substantial domestic practices [20]. This study was approved by the Institutional Review Board of the National Cancer Center Hospital in Tokyo, Japan (Approval Number 2021-476).

### Survey on the workload and quality control status of facilities

The workload study aimed to assess the changes in workload resulting from advancements in treatment techniques, calculate full-time equivalent (FTE) staffing based on case volume, determine the QC implementation ratio and evaluate the radiation management workload. Radiation management refers to the work required by national laws and regulations, such as receiving and discharging procedures for radioactive sources, documenting the time when radioactive sources are used, and patient entry and exit from radiation-controlled areas. We investigated the time RTMPs spent per case on each task, along with the implementation status and staffing adequacy for the QC of BT units. Detailed questions related to BT are provided in Supplementary Table 1. The FTE represents the workload ratio compared to the standard workload of a full-time employee, following the calculation method specified by the IAEA. The average working hours were adopted to evaluate the individual task workloads. The standard annual workload per staff member was set at 1760 h per year, considering a work schedule of 220 days per year and 8 h per day.

### Survey on the capabilities of individual radiotherapy technologists and medical physicists

This survey aimed to quantitatively investigate the skills of individual RTMPs to develop a future educational system and support the acquisition of qualifications. The survey inquired about the number of personnel who could perform each task related to patient irradiation and BT unit QC, as well as the individual abilities related to specific certifications. Comprehensive survey items related to BT are provided in Supplementary Table 2. Both the workload survey for facilities and the ability survey for individuals were limited to one response per respondent and did not collect patient identification information. We analyzed responses specifically from facilities where at least one BT case was conducted in the fiscal year 2022.

### Statistical analysis

All statistical analyses were performed using EZR, a statistical analysis software called R commander, plus statistical functions commonly used in biological and medical statistics [21]. Correlation coefficients were obtained using simple regression analysis. Kruskal–Wallis test followed by the Steel–Dwass test were used for multiple comparisons, with a *P*-value <0.05 considered statistically significant.

### Qualifications and professions related to radiotherapy in Japan

A radiation technologist is a person who irradiates the human body with radiation and can be engaged not only in radiotherapy but also in diagnostic radiology and nuclear medicine. A radiation technologist is the only national certification among the following, which is regulated by law in Japan, and is certified by completing a training school and passing a national examination.

A radiotherapy technologist (RT) is a generic term for those who are working to irradiate patients to radiation, but is not a specific qualification. Radiotherapy technologists must be certified as a radiation technologist. In Japan, the duties of a radiotherapy technologist also include acquiring CT images for treatment planning and implementing QC of radiotherapy equipment.

A qualified radiotherapy technologist (QRT) is a certified qualification granted to RTs who have been evaluated for their high level of expertise in radiation therapy through training and examinations conducted by the Japan Professional Accreditation Board for Radiotherapy Technologists, a general incorporated association.

A medical physicist (MP) is a healthcare professional who contributes from the perspective of medical physics to ensure the appropriate implementation of medical procedures involving radiation. The Japanese Board for Medical Physicist Qualification, a general incorporated association, conducts qualification examinations, and those who pass are certified. Many of the educational courses for MPs are offered as postgraduate courses at universities that train radiation technologists. Therefore, ~80% of MPs are also qualified as radiation technologists.

A qualified radiotherapy medical physicist (QMP) is a certified qualification granted to MPs who have demonstrated the ability to independently perform clinical medical physics tasks in radiation therapy at a high level. This qualification is evaluated and granted by the Japanese Board for Medical Physicist Qualification and is a higher qualification for MPs engaged in radiation therapy.

A radiotherapy quality manager (RQM) is a certified qualification granted to individuals with the ability and experience in quality management for the safe delivery of radiation therapy [22]. The Japanese Organization of Radiotherapy Quality Management, a voluntary organization, conducts the qualification examinations. One must qualify as an MP or a QRT to undertake the qualification examination.

## RESULTS

### Survey on the workload and quality control status of facilities

The questionnaire survey targeted all facilities that perform radiotherapy in Japan, and responses were received from 576 facilities. Of these, 124 facilities reported performing at least one case of GY-HDR, while 63 reported PR-LDR cases during the fiscal year 2022. In total, 45 facilities reported performing both GY-HDR and PR-LDR in at least one case. This corresponded to a previous report on responses from 85% and 66% of all domestic facilities performing HDR (146 facilities) and LDR (96 facilities), respectively [8]. Owing to the continuous decline in the usage of ^125^I seed sources, the actual number of facilities currently implementing PR-LDR may be less than the reported 96 facilities [23].

**Fig. 1:**
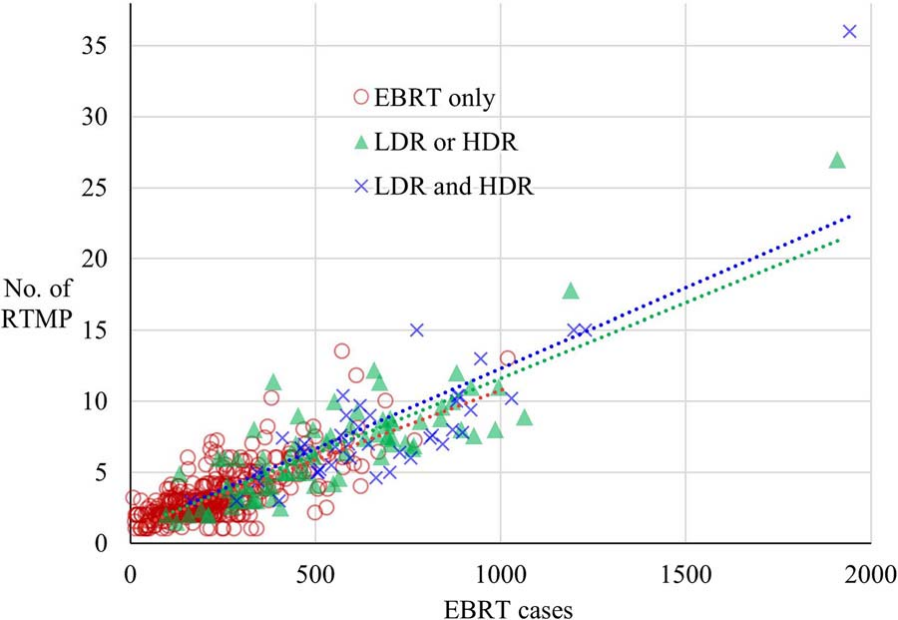
Number of EBRT cases and FTE RTMP personnel per facility, excluding particle therapy facilities. Facilities were categorized by the number of BT units they owned. The straight dotted lines indicate the results of a simple regression analysis. EBRT = external beam radiation therapy, FTE = full-time equivalent, RTMP = radiotherapy technologist plus medical physicist, HDR = high-dose-rate brachytherapy, LDR = low-dose-rate brachytherapy, No. = number.

The graph depicting the relationship between the number of EBRT cases per facility and the number of RTMP personnel expressed in FTE is presented in Fig. 1. Table 1 presents a summary of the data and the results of simple linear regression analysis. Particle therapy, including proton therapy, was excluded from these data because the time required for treatment planning and irradiation per patient is longer, leading to a discrepancy in the correlation between the number of RTMP personnel and the number of irradiated patients compared to X-ray equipment. Simple regression analysis was evaluated for all facilities and classified by the number of BT units owned by each facility. We adopted the value of 0.721 for the intercept of the simple linear regression line based on the number of BT units, which was consistent across all facilities. This choice accounts for the steep slope observed when BT unit numbers were high, leading to a reversal in RTMP FTEs with a small number of EBRT cases. We hypothesized that the minimum required RTMP FTE remains constant regardless of BT unit count. An increasing trend was observed between the number of BT units and both EBRT cases and RTMP FTEs. The slope in the simple linear regression analysis was steep.

**Table 1:** Summary of the simple regression analysis of the number of RTMP personnel and the number of EBRT cases evaluated by the number of BT units as shown in Fig. 1

	All	EBRT only	LDR or HDR unit	LDR and HDR unit
Respondents	518	406	80	42
EBRT median	241	209	492	632
RTMP median	3.0	3.0	6.3	7.2
Slope	0.01109	0.01064	0.01100	0.01165
Intercept	0.721	←	←	←
*R*	0.868	0.918	0.956	0.951
*P*-value	2.20E−16	2.30E−04	3.14E−04	5.86E−04

EBRT = external beam radiation therapy, RTMP = radiation technologist plus medical physicist, BT = brachytherapy, *R* = correlation coefficient.

**Fig. 2:**
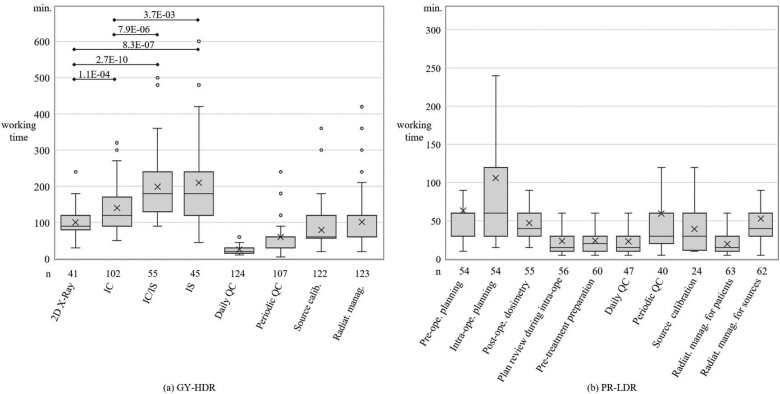
Boxplots of the working time obtained from the facility survey, (a) GY-HDR and (b) PR-LDR. The values in (a) GY-HDR indicate *P*-values by the Steel–Dwass test. 2D X-ray = two-dimensional planning using multi-directional X-ray images, IC = brachytherapy, IC/IS = intracavitary and interstitial brachytherapy, IS = interstitial brachytherapy, QC = quality control, Radiat. manag. = radiation management, ope. = operative. The values (*n*) listed below the horizontal axis represent the number of valid responses. Fig. 2. (a) Averaged working time in minutes: 101 (2D X-ray), 145 (IC), 211 (IC/IS), 210 (IS), 26 (Daily QC), 62 (Periodic QC), 80 (Source calibration), 102 (Radiation management). Fig. 2. (b) Averaged working time in minutes: 63 (Pre-ope. planning), 106 (Intra-ope. planning), 47 (Post-ope. dosimetry), 24 (Plan review during intra-ope.), 24 (Pre-treatment preparation), 23 (Daily QC), 59 (Periodic QC), 39 (Source calibration), 20 (Radiation management for patients), 53 (Radiation management for radioactive sources).

Figure 2 summarizes the working time required for each BT task. Specifically, the working time for GY-HDR was extended when performing 3D treatment planning using CT and MRI, in contrast to conventional 2D treatment planning utilizing bidirectional X-ray imaging. Notably, within the 3D treatment plans, the more complex IC/IS required longer working times than the IC. The results also revealed that a substantial amount of time was allocated to the QC of BT units and radiation management in accordance with legal and regulatory requirements. However, in the PR-LDR questionnaire, we did not inquire about the methods of intraoperative planning, which resulted in significant variability in the time spent on intraoperative planning.

The FTEs for each BT task, calculated from the responses to the questionnaire survey, are presented in Table 2. This table also shows the FTE calculations reported in other studies [14, 24–26] and the actual FTE obtained from a survey conducted in Oceania [27]. This FTE represents the combined RT and MP personnel, calculated by multiplying the average working time obtained from the survey by the number of personnel required for each task. This method is consistent with reports on EBRT [18] and report from IAEA [14]. The number of RTMP personnel required for each task was determined in consultation with a co-author with extensive experience in BT. For GY-HDR, the patient-dependent calculation was based on two RTMP personnel multiplied by each treatment method.

**Table 2:** FTE factors calculated from the averaged working hours per task; working hours were obtained from the facility questionnaire

		FTE calculation factor					Actual FTE from survey responses			
		EC [24]	IPEM [25]	COMP [26]	IAEA [14]		ACPSEM [27]		This study	
	Occupation	RT + MP	RT + MP	RT + MP	RT + MP		MP		RT + MP	
	*Patient dependent*									
**HDR**	Per fraction	0.0027^*^	0.0027^*^	0.0025	0.0020	(2D)	N/A		0.0019	(2D)
					0.0029	(3D)	0.0020	(Simple IC)	0.0028	(3D)
							0.0031	(Complex IC)	0.0040	(IC/IS)
					0.0037	(IS)	0.0028	(IS)	0.0039	(IS)
	*Equipment dependent*									
	HDR unit	0.4	0.4	0.15	0.15		0.060		0.045	
	TPS	0.2	0.4	0.15	0.03		0.032		Inclu.	
	Others	N/A	N/A	0.32	0.18		0.000		Inclu.	
	CT simulator	0.4	0.4	0.15	0.07		0.031		0.017	
	*EQP total*	*1.0*	*1.2*	*0.77*	*0.43*		*0.122*		*0.063*	
**LDR**	*Patient dependent*									
	Per patient	0.008	0.0081	0.0025	0.004		0.0024		0.0035	
	*Equipment dependent*									
	LDR unit	0.4	0.1	0.08	0.15		0.011		0.018	
	TPS	0.2	0.2	0.15	0.03		0.032		Inclu.	
	Others	N/A	0.1	0.08	0.18		0.008		Inclu.	
	*EQP total*	*0.6*	*0.4*	*0.31*	*0.36*		*0.050*		*0.018*	

The values marked with an asterisk (^*^) were originally reported per patient in the literature but have been adjusted by dividing by the standard GY-HDR fractionation number of 3 for comparison with other data.

FTE = full-time equivalent, EC = European Commission, IPEM = Institute of Physics & Engineering in Medicine, COMP = Canadian Organization of Medical Physicists, IAEA = International Atomic Energy Agency, ACPSEM = Australasian College of Physical Scientists and Engineers in Medicine, RT = radiotherapy technologist, MP = medical physicist, HDR = high dose rate, LDR = low dose rate, fxs = fractions, 2D = two-dimensional image-based treatment planning, 3D = three-dimensional image-based treatment planning, IC = intracavitary brachytherapy, IC/IS = intracavitary and interstitial brachytherapy, IS = interstitial brachytherapy, TPS = treatment planning system, Other = dosimetric equipment and ultrasound, among others, EQP = equipment, N/A = indicates responses that were not available or could not be referenced in the literature. Inclu. = notation in the column for this study indicates that the FTE calculation for the BT units included the treatment planning system and dosimetric equipment.

Italicized entries indicate the classification of the FTE calculation.

Similarly, for equipment-dependent tasks, considering the potential impact on the overall treatment of patients, two RTMP personnel were assigned for periodic QC and source strength calibration. In contrast, daily QC and radiation management were assigned to one person. For PR-LDR, the number of RTMP personnel required for each task was determined as follows: one person for patient-dependent tasks, including patient irradiation (from preoperative planning to plan review during intraoperative procedures). This is because, in Japan, these tasks are often performed as an adjunct to radiation oncologists or urologists. For equipment-dependent tasks, two personnel were assigned for periodic QC and source strength calibration, considering their potential impact on the overall treatment of patients. Daily QC and radiation management were assigned to one person.

**Fig. 3:**
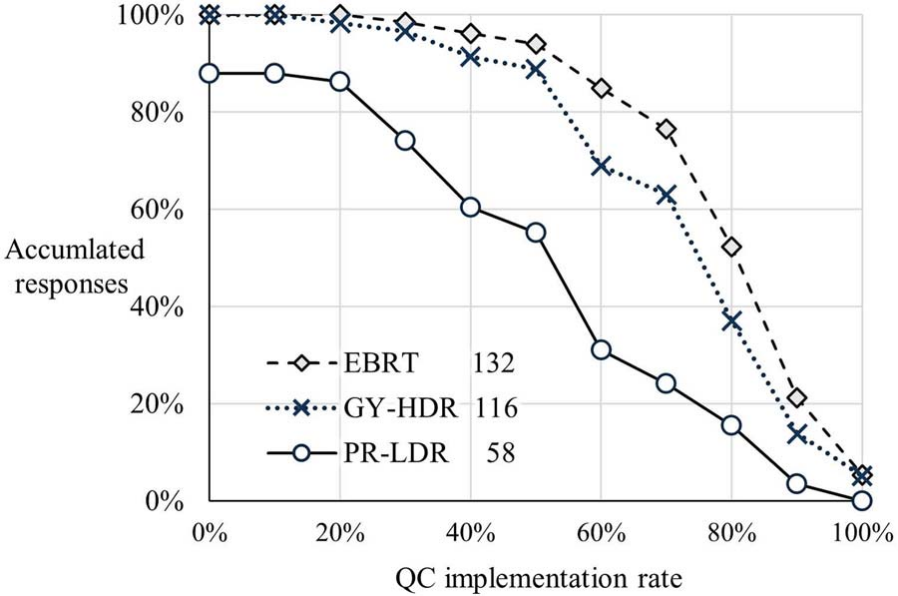
Cumulative implementation rate of QC items according to the guidelines based on responses. The EBRT data were restricted to facilities with at least one GY-HDR or PR-LDR case in fiscal year 2022. The numerical values adjacent to the legend items indicate the corresponding number of survey respondents. For all combinations, statistically significant differences were detected at a significance level of *P* <0.05 using the Steel–Dwass test. EBRT = external beam radiation therapy, GY-HDR = gynecological cancer treated with high-dose-rate brachytherapy, PR-LDR = permanent prostate implantation using ^125^I low-dose-rate brachytherapy, QC = quality control.

**Fig. 4:**
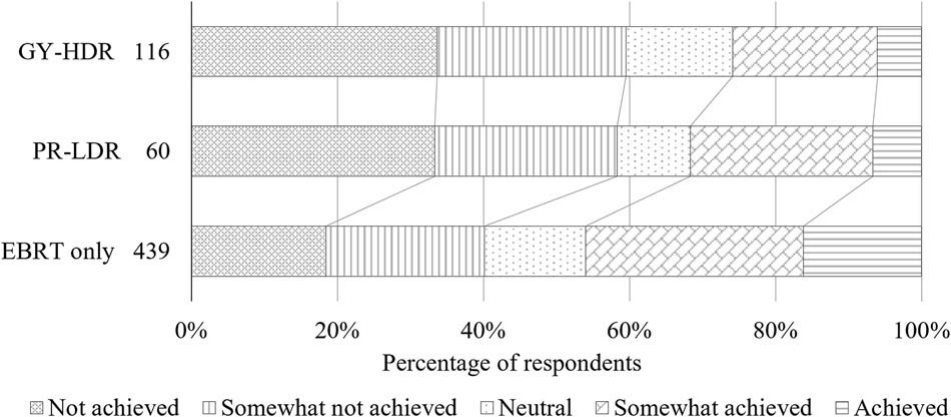
Fulfillment of QC work duties within regular working hours. The numerical values adjacent to the legend items indicate the corresponding number of survey respondents. For both GY-HDR and PR-LDR compared to EBRT, significant differences were observed at a significance level of *P* <0.05 using the Steel–Dwass test. However, no significant difference was found between GY-HDR and PR-LDR. GY-HDR = gynecological cancer treated with high-dose-rate brachytherapy, PR-LDR = permanent prostate implantation using ^125^I low-dose-rate brachytherapy, EBRT = external beam radiation therapy, QC = quality control.

Figure 3 shows the status of QC implementation in BT units. The horizontal axis is the achievement rate of the items recommended by the national guidelines [6], and the vertical axis is the cumulative number of responding facilities. The EBRT data shown in Fig. 3 are limited to responses from facilities performing at least one case of either GY-HDR or PR-LDR. This evaluation aimed to determine the prioritization of QC for equipment in facilities offering both BT and EBRT. Specifically, the implementation rate of QC items for BT units, as outlined in the guidelines, was notably low for PR-LDR. Overall, 12% of the facilities did not implement QC of the PR-LDR units. Figure 4 shows the proportion of QC tasks accomplished during regular working hours, and Table 3 presents the percentage of personnel expressing the need for increased engagement of RTMPs in QC. Notably, the facilities performing GY-HDR or PR-LDR exhibited lower implementation rates during regular working hours than those that did not. It was found that the QC of BT units tends to be conducted outside of regular working hours. Facilities providing BT expressed a greater need for additional personnel involvement in QC.

### Survey on the capabilities of individual radiotherapy technologists and medical physicists

**Fig. 5:**
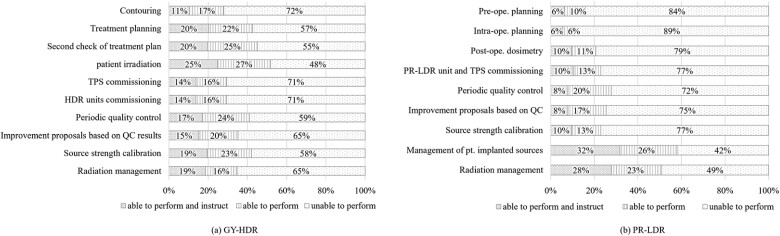
Percentage of respondents who answered, ‘able to perform and instruct’ and ‘able to perform’ in each BT task, (a) GY-HDR (*n* = 745), (b) PR-LDR (*n* = 247). GY-HDR = gynecological cancer treated with high-dose-rate brachytherapy, PR-LDR = permanent prostate implantation using low-dose-rate brachytherapy, BT = brachytherapy, QC = quality control, TPS = treatment planning system, HDR = high dose rate.

The questionnaire for RTMP individuals received 2307 responses from 699 facilities. Specifically, the data analyzed in this study were restricted to responses from facilities conducting at least one case of either GY-HDR or PR-LDR in the fiscal year 2022. In total, 745 and 384 responses were obtained for the GY-HDR and PR-LDR groups, respectively. The results of the individual capabilities in BT procedures and radiation management, categorized into three options: ‘able to perform and instruct’, ‘able to perform’ and ‘unable to perform’, are shown in Fig. 5. The definition of ‘able to instruct’ is the ability to educate newly assigned RTMPs. These criteria were selected based on the respondents’ self-judgment. In GY-HDR, the proportion of RTMPs who were able to perform tasks related to treatment planning, patient dose delivery and plan check was higher than that in PR-LDR. These are directly related to the workflow of patient treatment. Conversely, for PR-LDR, the overall proportion of respondents who were unable to perform was higher than that for GY-HDR. Even among RTMPs engaged in facilities performing BT, their ability to perform QC for BT units was significantly lower (approximately <40% for GY-HDR and <20% for PR-LDR), which is markedly below the ~80% for EBRT [18].

**Fig. 6:**
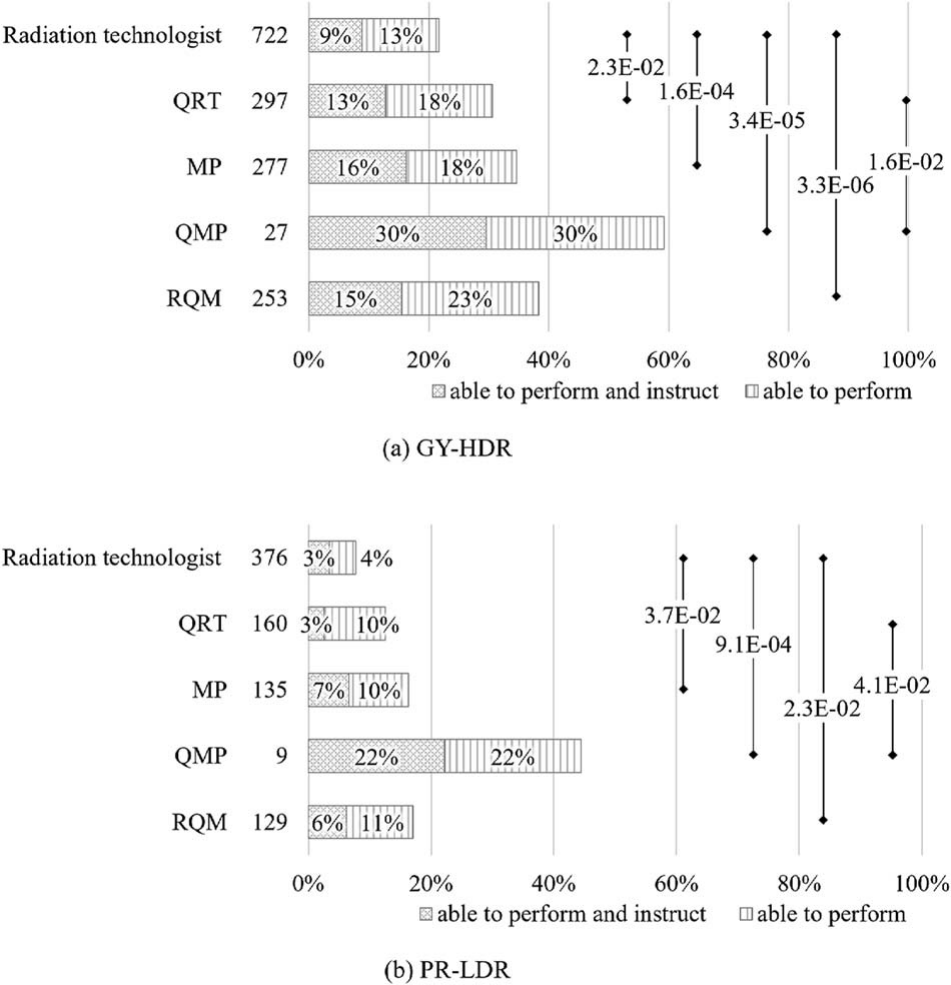
Percentage of respondents who answered, ‘able to perform and instruct’ and ‘able to perform’ based on their certification qualifications. From the items in Fig. 5, (a) GY-HDR refers to those who can perform all tasks except for radiation management, contouring and patient irradiation. (b) PR-LDR includes individuals who can perform all tasks except for pre-to-post treatment planning and radiation management. The numbers next to the qualifications indicate the corresponding number of survey respondents. GY-HDR = gynecological cancer treated with high-dose-rate brachytherapy, PR-LDR = permanent prostate implantation using low-dose-rate brachytherapy, RT = radiotherapy technologists, QRT = qualified radiotherapy technologist, MP = medical physicist, QMP = qualified radiotherapy medical physicist, RQM = radiotherapy quality manager.

Figure 6 shows the differences in the ability to perform and instruct according to the qualifications held. The results summarize the percentages of respondents who answered ‘able to perform and instruct’ or ‘able to perform’ for all items considered necessary for RTMPs. However, this evaluation excluded (a) items related to radiation management, contouring and patient irradiation for GY-HDR and (b) items related to pre-to-post treatment planning and radiation management for PR-LDR. In Japan, the involvement of RTMPs in these tasks is limited or restricted by law to specific professions. Compared to RT, those with higher qualifications, such as QRT and QMP, tended to have higher percentages of respondents who answered ‘able to perform and instruct’ or ‘able to perform’.

## DISCUSSION

Through the comprehensive national survey conducted in this study, we performed a quantitative evaluation of the workload and individual technical skills of RTMPs in BT in Japan. Additionally, we identified issues related to the working environment and education systems of RTMPs engaged in BT, which we believe will provide useful information not only for Japan but also for developing countries where IGBTs will spread in the future.

Findings from a facility-based survey in Fig. 1 revealed a strong correlation between the number of patients with EBRT and RTMP personnel. In a previous survey conducted at designated cancer care hospitals, the number of patients receiving EBRT demonstrated a significant correlation with the FTE personnel of RT [28, 29]. In this study, the regression coefficients of the number of patients with EBRT and RT personnel expressed in FTEs were evaluated according to the number of BT units owned by the facilities (Table 1). It was evident that the greater the number of BT units, the larger the regression coefficient of the linear regression equation, indicating that personnel are allocated for BT-related tasks. Conversely, in the survey by Giddings *et al*. targeting RTs [30], the number of responding facilities was small (36), resulting in no difference in FTE values depending on the implementation status of BT and/or stereotactic radiosurgery. However, in this study, the large number of responding facilities (576) revealed these differences. Thus, we need to evaluate and reflect the workload based not only on the number of BT units but also on the number of BT patients [31], the number of personnel required for appropriate QC, the BT techniques performed at the facility and the workload of radiation management tasks to calculate the necessary number of RTMP personnel.

In GY-HDR, the working time of the RTMPs was prolonged as the technical complexity escalated from 2D to 3D and from IC to IC/IS and IS techniques, as depicted in Fig. 2. This elongation is attributed to factors such as the time required for 3D treatment planning, difficulty in needle placement inside the tissue in IS (including IC/IS) and the increased frequency of X-ray fluoroscopy [32]. Furthermore, the utilization of 3D fluoroscopy with CT is recognized as a contributing factor to the increased RTMP workload [33]. A previous nationwide survey on GY-HDR, conducted via a questionnaire, indicated that the time utilized by RTMP was 73 min in 2D procedures and 94 min in 3D procedures, which was less than the results of the current study [32]. Our data collection spanned from patient entry to exit, capturing the working hours per task. This approach differs from previous reports that focused on specific tasks (e.g. treatment planning and image acquisition). Additionally, even when not planning treatment or acquiring images, RTMPs have duties such as pre-checking patient treatment plans, registering treatment records in the RIS and monitoring the room camera during applicator insertion. Another contributing factor is the widespread adoption of IGBT using MRI, which entails a higher workload than CT. Notably, in Japan, the proportion of facilities implementing 3D IGBT has steadily increased from 16% in 2012 [34] to 48% in 2016 [32] and further to 74% in 2022 [8]. Moreover, the adoption of GY-HDR using IC/IS techniques is projected to increase owing to enhanced dose distribution relative to IC and the establishment of domestic guidelines [35, 36]. Therefore, the workload required for RTMPs in GY-HDR is expected to continue increasing. The PR-LDR involves significant variations in responsibilities across facilities. For instance, urologists and radiation oncologists are primarily responsible for creating treatment plans, including intraoperative planning. RTMPs often focus on tasks mandated by domestic regulations, such as managing required radiation sources and searching for missing or dropped seeds. Therefore, in PR-LDR scenarios, RTMPs may inadvertently underestimate or overlook their workload because they are frequently not directly engaged in patient treatment.

Furthermore, the annual volume of cases per facility varied considerably. While the median was 13 cases, eight facilities exceeded 60 cases per year [8]. Therefore, the appropriate workload for both the GY-HDR and PR-LDR should be assessed and reflected in the staffing numbers of the facilities.

The survey results indicate that the actual FTE workload related to patient treatment for BT is comparable to the survey responses from Oceania [27] and other reports [24–26], including that from the IAEA [14], as summarized in Table 2. Conversely, Japan’s involvement in equipment-dependent tasks that constitute the QC of BT units is notable. Both GY-HDR and PR-LDR exhibited workload levels of less than half the measured values in Oceania. Additionally, the FTE value was significantly lower than those values reported by the other studies [14, 24–26]. One factor contributing to the low FTE values calculated in this study is considered to be the insufficient implementation of BT QC. Figure 3 shows whether the QC of BT units has been properly implemented in Japan.

Regarding the implementation rate of QC items of radiotherapy equipment according to the guidelines, BT had lower rates than EBRT, particularly for PR-LDR. Figure 4 illustrates that facilities performing BT require more time to complete QC, exceeding the regular working hours. This may be associated with the shorter engagement time for the BT QC unit. An analogous survey targeting MPs in Oceania also reported a strong desire to allocate more time to BT-related tasks [27]. As shown in Table 3, there is a particular need for more RTMPs to be engaged in QC in facilities at facilities performing BT. These results suggest that the low implementation rate of QC for BT units, as shown in Fig. 3, is due to a shortage of RTMP personnel.

**Table 3:** Proportion of respondents who expressed a desire for an increased number of RTMP personnel engaged in QC, classified based on facilities performing EBRT only and each type of BT

Respondent facilities	Responses	Desired	Desired %
EBRT only	509	301	59
GY-HDR > 1 case	116	94	81
PR-LDR > 1 case	60	53	88

GY-HDR = gynecological cancer treated with high-dose-rate brachytherapy, PR-LDR = permanent prostate implantation using ^125^I low-dose-rate brachytherapy, BT = brachytherapy, EBRT = external beam radiation therapy, QC = quality control.

Additionally, as shown in Fig. 4, the workload for RTMPs engaged in BT is high because QC tasks cannot be fulfilled during regular working hours. Several factors need to be considered as follows. First, there has been a recent shift in workload toward external beam radiation. The Japanese structure survey of radiation oncology conducted by JASTRO reported that in 2009, the FTE for RTMPs was 4.32, with a total of 4296 intensity-modulated radiation therapy (IMRT) cases [37]. Ten years later, by 2019, the FTE for RTMPs increased to 6.06, while the total number of IMRT cases increased ninefold to 39 941. Tohyama *et al*. reported that the patient-dependent FTE for IMRT was approximately twice that of three-dimensional conformal radiation therapy (3DCRT) [18].

Additionally, ensuring the quality of dose distribution in IMRT requires more advanced QC of EBRT equipment. Thus, the increased workload for EBRT has likely led to the postponement of QC for BT units. Second, because there are many facilities capable of performing BT in Japan, the number of personnel engaged in BT and the importance of QC at these facilities differ from those in other developed countries, where each facility treats >100 BT patients [38–40]. Another reason is that reimbursement for QC of radiotherapy equipment is only recognized for EBRT, leading to a low implementation rate of QC for BT units. Therefore, it can be inferred that the QC of BT units in Japan is insufficient due to a lack of personnel and other factors, resulting in structural issues. To solve these problems, the radiotherapy department needs to recognize the importance of QC for BT units handling radiation sources and establish a system that can implement it. To establish such a system, it is essential to determine the number of RTMPs based on an appropriate evaluation of BT workload, as shown in Table 2, and also to establish an appropriate educational system. Additionally, it should be considered to reflect QC for BT units in reimbursement, similar to EBRT.

Although the responses depicted in Fig. 5 were limited to RTMPs working at facilities that implemented GY-HDR or PR-LDR, except for those that implemented EBRT only, substantially few RTMPs answered ‘able to perform’ and ‘able to perform and instruct’. Particularly, the high rate of ‘unable to perform’ for PR-LDR was notable. In Japan, the increase in MPs in the late 2000s, along with the establishment of certification systems such as for QRTs and RQMs, has spread the importance of commissioning and QC in radiation therapy. Since PR-LDR began in the mid-2000s, before these periods, the practice was established by radiation oncologists and urologists, with RTMPs tending to engage in related radiation management. This situation has continued, leading to a tendency for RTMPs to be less actively involved in treatment planning and QC for PR-LDR. Thus, due to the limited number of personnel who can perform BT-related tasks, it is expected that the workload is concentrated on the RTMPs who can perform these tasks.

Consequently, this may result in a significant burden on specific RTMPs. This factor might contribute to why QC is not completed during regular working hours, and the implementation rate of QC in accordance with the guidelines is low. Ensuring safe BT for patients necessitates the expertise of a ‘generalist’ who possesses broad knowledge and can identify equipment malfunctions and treatment planning errors. BT generalists who comprehend the entire BT workflow, including treatment planning, BT unit commissioning and periodic QC, should actively participate in patient care. We strongly advocate having at least one BT generalist with these capabilities at each facility. Furthermore, based on insights gained from previous BT incidents, it is essential to establish backup personnel capable of transferring knowledge and skills [9].

Figure 6 shows a positive correlation between the certification level and RTMPs; as the certification level increases, the proportion of RTMPs capable of both performing and instructing increases. Therefore, individuals with advanced certifications are expected to demonstrate enhanced practical BT skills. QMPs specializing in radiation therapy are considered to have practical competencies. However, since the QMP system was established in 2019 and the number of certified individuals is still limited, it is necessary to monitor whether this trend continues. As shown in Fig. 5, both GY-HDR and PR-LDR demonstrate a significant shortage of skilled personnel for commissioning tasks. A potential scenario exists in which no individual within a facility can perform these tasks. Organizing workshops or training sessions that focus on practical techniques for enhancing knowledge and skills is necessary to address these challenges [41, 42].

However, this study has certain limitations. BT procedures, such as accelerated partial breast irradiation with HDR, ophthalmic affixation treatment with ^106^Lu, and the use of needles and hairpins for LDR, were excluded. This exclusion was attributed to the limited availability of facilities performing these procedures and the significant variations in techniques across different facilities. Workload assessment does not account for tasks related to acceptance tests, commissioning and other activities associated with equipment upgrades, which are typically conducted every 10–15 years. These tasks require experienced and knowledgeable RTMPs to allocate sufficient time to ensure a safe BT. Our calculated FTE for BT did not include networking, such as integrating radiological information systems and treatment planning systems. Moreover, it did not account for the time overlap with EBRT, including administrative duties and staff training. Notably, the FTEs calculated in this study are likely to underestimate the true workload of the RTMPs.

In conclusion, we conducted a survey on the working environment and QC status of RTMPs engaged in GY-HDR and PR-LDR within Japan. Recently, in GY-HDR, the increasing complexity and high knowledge requirements associated with 3D IGBT and IC/IS have significantly increased the workload of RTMP beyond the traditional 2D BT level. Compared to reports from other countries, the FTE workload related to patient treatment aligns closely. Nevertheless, the QC status of BT units continues to lag significantly in terms of both working hours and implementation rates. Moreover, our findings revealed that the number of individuals capable of performing BT tasks within facilities is limited, suggesting that few RTMPs bear disproportionate workloads. Based on these findings, we concluded that the personnel system for implementing QC of BT units domestically is insufficient. Consequently, we recommend raising awareness regarding its importance, integrating it into reimbursement systems akin to EBRT, and offering practical educational programs to enhance the current state of BT unit QC. The issues identified in this study may also arise in developing countries. We believe that our future work addressing these issues will provide helpful information for safe BT worldwide.

## Supplementary Material

08_SupplementalData_noChange_rrae082

## Data Availability

The datasets generated and/or analyzed in the current study are available from the corresponding author upon reasonable request.

## Presentation at a conference

Partial results of this study were presented at the 25th annual meeting of the Japanese Group of Brachytherapy/Japanese Society for Radiation Oncology, Kobe, 2023.
