# Supplementary material for: Current status of the working environment of brachytherapy in Japan: a nationwide survey-based analysis focusing on radiotherapy technologists and medical physicists
Source: J Radiat Res. 2024 Oct 24;65(6):851–61. doi: 10.1093/jrr/rrae082 (PMC11629993; doi:10.1093/jrr/rrae082)
Supplement: 08_SupplementalData_noChange_rrae082 [file 08_supplementaldata_nochange_rrae082.docx]

Supplement Table 1. Survey questions on the workload and QC status for brachytherapy facilities. Only the questions related to brachytherapy in this study are presented from the overall questions. All questions were conducted in Japanese.

| Questions about the status of the facility’s operations. |
| --- |
| - The number of RTMPs in charge of radiotherapy. - Number of new patients and re-irradiated patients for EBRT, excluding particle therapy and gamma knife therapy. Of these, the number of patients who received conventional irradiation and IMRT, respectively. - Number of GY-HDR and PR-LDR patients. - Implementation rate of QC according to the guidelines in EBRT, GY-HDR, and PR-LDR. - Fulfillment of QC work duties within regular working hours. |
| Questions about workload for GY-HDR (unit: minute). |
| - Two-dimensional planning brachytherapy: Time per irradiation from patient entry to exit. - Three-dimensional intracavitary: Time per irradiation from patient entry to exit. - Three-dimensional intracavitary and interstitial: Time per irradiation from patient entry to exit. - Three-dimensional interstitial: Time per irradiation from patient entry to exit. - Monthly periodic QC of HDR unit. - Other QC time for treatment equipment per month. - In-house source calibration of HDR sources per session. - Source ordering, commissioning, registration, shipping, and form preparation regulated by domestic law per session. |
| Questions about workload for PR-LDR (unit: minute). |
| - Pre-operation treatment planning for determining the number of sources ordered. - Intra-operation treatment planning. - Post-operation patient dosimetry. - Review and approval of intra-operation planning. - Daily QC and preparation for PR-LDR operation, including set-up of X-ray, ultrasound, seed sources, and other equipment. - Monthly periodic QC of PR-LDR unit. - Other QC time for treatment equipment per month. - In-house seed source calibration per session. - Radiation management such as searching for missing seed sources and dosimetry at patient exit from radiation-controlled area per session. - Source ordering, commissioning, registration, shipping, and form preparation regulated by domestic law per session. |

Supplement Table 2. Questions of the survey on the capabilities of individual RTMPs working in brachytherapy. The respondents were asked to choose from the following three options: “able to perform and instruct,” “able to perform,” or “unable to perform.”

| Questions about personal skills for GY-HDR. | |
| --- | --- |
| Treatment planning | - Contouring for organs at risk. - Applicator reconstruction and optimization of dose distribution. - Plan review and approval. - Patient dose delivery. |
| QC of HDR unit | - Commissioning of treatment planning system of HDR. - Commissioning of HDR unit. - Periodic QC in accordance with guidelines. - Propose necessary measures for quality improvement based on the results of QC of treatment equipment. - In-house source calibration of HDR source. - Source ordering, commissioning, registration, shipping, and form preparation regulated by domestic law per session. |
| Questions about personal skills for PR-LDR. | |
| Treatment planning | - Pre-operation treatment planning for determining the number of sources ordered. - Intra-operation treatment planning. - Post-operation patient dosimetry. |
| QC of HDR unit | - Commissioning of PR-LDR unit, including treatment planning system. - Periodic QC in accordance with guidelines. - Propose necessary measures for quality improvement based on the results of QC of treatment equipment. - In-house seed source calibration. - Radiation management such as searching for missing seed sources and dosimetry at patient exit from radiation-controlled area. - Source ordering, commissioning, registration, shipping, and form preparation regulated by domestic law. |
